# Comparing the xylose reductase/xylitol dehydrogenase and xylose isomerase pathways in arabinose and xylose fermenting *Saccharomyces cerevisiae *strains

**DOI:** 10.1186/1754-6834-1-16

**Published:** 2008-10-23

**Authors:** Maurizio Bettiga, Bärbel Hahn-Hägerdal, Marie F Gorwa-Grauslund

**Affiliations:** 1 Department of Applied Microbiology, Lund University, PO Box 124, SE-22100 Lund, Sweden

## Abstract

**Background:**

Ethanolic fermentation of lignocellulosic biomass is a sustainable option for the production of bioethanol. This process would greatly benefit from recombinant *Saccharomyces cerevisiae *strains also able to ferment, besides the hexose sugar fraction, the pentose sugars, arabinose and xylose. Different pathways can be introduced in *S. cerevisiae *to provide arabinose and xylose utilisation. In this study, the bacterial arabinose isomerase pathway was combined with two different xylose utilisation pathways: the xylose reductase/xylitol dehydrogenase and xylose isomerase pathways, respectively, in genetically identical strains. The strains were compared with respect to aerobic growth in arabinose and xylose batch culture and in anaerobic batch fermentation of a mixture of glucose, arabinose and xylose.

**Results:**

The specific aerobic arabinose growth rate was identical, 0.03 h^-1^, for the xylose reductase/xylitol dehydrogenase and xylose isomerase strain. The xylose reductase/xylitol dehydrogenase strain displayed higher aerobic growth rate on xylose, 0.14 h^-1^, and higher specific xylose consumption rate in anaerobic batch fermentation, 0.09 g (g cells)^-1 ^h^-1 ^than the xylose isomerase strain, which only reached 0.03 h^-1 ^and 0.02 g (g cells)^-1^h^-1^, respectively. Whereas the xylose reductase/xylitol dehydrogenase strain produced higher ethanol yield on total sugars, 0.23 g g^-1 ^compared with 0.18 g g^-1 ^for the xylose isomerase strain, the xylose isomerase strain achieved higher ethanol yield on consumed sugars, 0.41 g g^-1 ^compared with 0.32 g g^-1 ^for the xylose reductase/xylitol dehydrogenase strain. Anaerobic fermentation of a mixture of glucose, arabinose and xylose resulted in higher final ethanol concentration, 14.7 g l^-1 ^for the xylose reductase/xylitol dehydrogenase strain compared with 11.8 g l^-1 ^for the xylose isomerase strain, and in higher specific ethanol productivity, 0.024 g (g cells)^-1 ^h^-1 ^compared with 0.01 g (g cells)^-1 ^h^-1 ^for the xylose reductase/xylitol dehydrogenase strain and the xylose isomerase strain, respectively.

**Conclusion:**

The combination of the xylose reductase/xylitol dehydrogenase pathway and the bacterial arabinose isomerase pathway resulted in both higher pentose sugar uptake and higher overall ethanol production than the combination of the xylose isomerase pathway and the bacterial arabinose isomerase pathway. Moreover, the flux through the bacterial arabinose pathway did not increase when combined with the xylose isomerase pathway. This suggests that the low activity of the bacterial arabinose pathway cannot be ascribed to arabitol formation via the xylose reductase enzyme.

## Background

Ethanol produced by fermentation of plant biomass is considered to be an environmentally friendly alternative to fossil fuels [1-3]. Cost-effective and sustainable production of ethanol as a transportation fuel entails the utilisation of microbial strains able to ferment completely all the sugars in lignocellulosic hydrolyzates [4-6]. Baker's yeast *Saccharomyces cerevisiae*, which has been used for ethanol production since the beginning of history [7], displays efficient ethanolic fermentation of sugar and starch-based raw materials. The selection process has also made *S. cerevisiae *a very robust organism, which tolerates high ethanol concentrations and is able to cope with harsh environments [8]. However, *S. cerevisiae *is unable to utilise arabinose and xylose, which in some raw materials such as agricultural residues and hardwoods, can account for more than 30% of total sugars [9], and which constitutes a significant barrier to the cost-effectiveness and sustainability of bioethanol production [6]. *S. cerevisiae *has been extensively engineered, developed and adapted to expand its substrate range to include the utilisation of the pentose sugars, arabinose and xylose, for growth and ethanol production [4].

**Figure 1:**
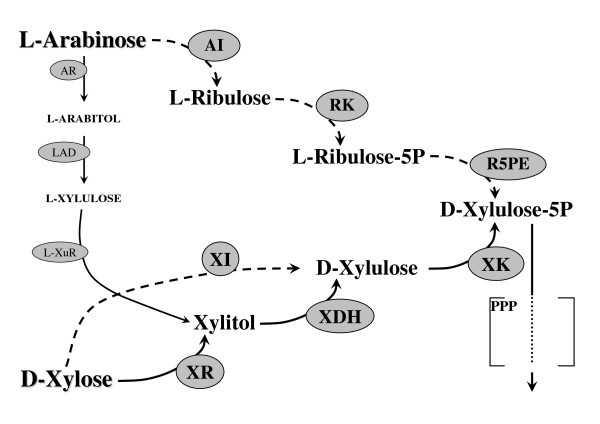
**Xylose and arabinose utilisation pathways**. Solid lines: oxidation/reduction-based pathways; dashed lines: isomerisation-based pathways. PPP: pentose phosphate pathway. AI: arabinose isomerase; AR: arabinose reductase; LAD: arabitol dehydrogenase; L-XuR: L-xylulose reductase; R5PE: ribulose-phosphate-5-epimerase; RK: ribulokinase; XDH: xylitol dehydrogenase; XI: xylose isomerase; XK: xylulokinase; XR: xylose reductase.

To enter the central carbon metabolism, arabinose and xylose must first be converted to xylulose 5-phosphate, an intermediate compound of the pentose phosphate pathway (PPP) (Figure 1). Essentially, two different pathways are available in nature for the conversion of pento-aldoses to xylulose: reduction/oxidation-based pathways and isomerisation-based pathways.

In bacteria, L-arabinose is converted to L-ribulose, L-ribulose-5-P and finally D-xylulose-5-P via L-arabinose isomerase (AraA) [10-13], L-ribulokinase (AraB) [10,11,13,14] and L-ribulose-5-P 4-epimerase (AraD) [10,11,13,15], respectively (Figure 1). In fungi, L-arabinose is reduced to L-arabitol by arabinose reductase [16]. Arabitol is then re-oxidised by arabitol dehydrogenase to give L-xylulose [17], which is in turn converted to xylitol by L-xylulose reductase [17]. Xylitol is finally converted to xylulose by xylitol dehydrogenase (XDH) [18,19], whose activity is also part of xylose utilisation pathways (Figure 1).

In pentose-growing yeasts, xylose is first reduced by xylose reductase (XR) to xylitol [20], which in turn is oxidised to xylulose by XDH (Figure 1) [18,19]. In bacteria and some anaerobic fungi, xylose isomerase (XI) is responsible for direct conversion of xylose to xylulose [21-23] (Figure 1). Xylulose is finally phosphorylated to xylulose-5-phosphate by xylulokinase (XK) [24]. In *S. cerevisiae*, pentose sugar fermentation has been achieved by introducing several different alternative pathways, recently reviewed in Hahn-Hägerdal et al [25].

In the present investigation, we compared two xylose and arabinose co-consuming *S. cerevisiae *strains, which expressed two different xylose utilisation pathways and were otherwise genetically identical. A strain expressing XR and XDH and harbouring a bacterial arabinose utilisation pathway was compared with an isogenic strain instead expressing the XI xylose utilisation pathway. Pentose utilisation was characterised with respect to aerobic arabinose or xylose growth. Substrate consumption and product formation during anaerobic co-fermentation of glucose, arabinose and xylose was also investigated. The XR/XDH strain displayed faster aerobic growth on xylose and outperformed the XI strain with respect to pentose sugar consumption and ethanol production.

## Results

### Construction of arabinose and xylose fermenting strains TMB3075 (XR/XDH strain) and TMB3076 (XI strain)

Two strains, harbouring a chromosomally integrated, bacterial arabinose utilisation pathway [26] that consists of L-arabinose isomerase (*Bacillus subtilis AraA*), L-ribulokinase (*Escherichia coli AraB*) and L-ribulose-5-phosphate 4-epimerase (*E. coli AraD*) [26,27], in combination with two different plasmid-borne xylose pathways, were constructed. The two strains, which contained either the XR/XDH pathway or the XI pathway (Figure 1), will be referred to as the 'XR/XDH strain' and 'XI strain', respectively (Table 1). The XR/XDH strain harbours the arabinose pathway in combination with *P. stipitis XYL1 *and *XYL2 *genes encoding XR and XDH, respectively [28]. For the XI strain, the same arabinose pathway is combined with the *Piromyces *XI gene (*xylA*) [29,30].

**Table 1:** Plasmids and strains used in this study

**Plasmid**	**Features**	**Reference**
prDNAAraA	pBluescript, *NTS2::pHXT7*_*tr*_*-AraA(B. subtilis)-tCYC1*	[27]
prDNAAraD	pBluescript, *NTS2::pHXT7*_*tr*_*-AraD (E. coli)-tCYC1*	[27]
pY7	*XYL1 (P. stipitis), XYL2 (P. stipitis), URA3*	[28]
YEplacHXT-XIp	*pHXT7*_*tr*_*-*XI *(Pyromyces sp.)-tCYC1, URA3*	[29]
YIpAraB	*KanMX, pHXT7*_*tr*_*-AraB (E. coli)-tCYC1, TRP1*	[27]
YIplac128	*LEU2*	[53]
		
**Strain**	**Genotype**	**Reference**
TMB3042	CEN.PK 2-1C, *Δgre3, his3:: p PGK1-XKS1- t PGK1, TAL1:: p PGK1-TAL1- t PGK1, TKL1:: p PGK1-TKL1- t PGK1, RKI1:: p PGK1-RKI1- t PGK1, RPE1:: p PGK1-RPE1- t PGK1, leu2, trp1, ura3*	[31]
TMB3070	TMB3042, YIpAraB	This work
TMB3073	TMB3070, *pHXT7*_*tr*_*-AraA(B. subtilis)-tCYC1, NTS2::pHXT7*_*tr*_*-AraD (E. coli)-tCYC1*	This work
TMB3074	TMB3073, YIplac128	This work
TMB3075 (xylose reductase/xylitol dehydrogenase strain)	TMB3074, pY7	This work
TMB3076 (xylose isomerase strain)	TMB3074, YEplacHXT-XIp	This work

The starting strain TMB3042 (Table 1) harbours genetic modifications previously reported to be favourable for pentose fermentation, such as overexpression of PPP [31] and XK [32] (Figure 1) and deletion of *GRE3*, encoding an endogenous unspecific aldose reductase (AR) [33]. Gre3p, like XR, is able to reduce xylose to xylitol, but with the exclusive use of nicotinamide adenine dinucleotide phosphate (NADPH) as a cofactor [34]. Removal of the NADPH-dependent activity of Gre3p is therefore, beneficial for the cofactor balance of a strain expressing *P. stipitis *XR (*XIL1*), which can perform the reduction also using nicotinamide adenine dinucleotide (NADH) [31,35]. In addition, xylitol is a known inhibitor of XI [36] and deletion of *GRE3 *in an XI strain prevents the formation of this compound and has therefore, a beneficial effect on xylose fermentation [33,37].

First, the bacterial arabinose utilisation pathway (Figure 1) was integrated in TMB3042. The gene *AraB *from *E. coli *was integrated in a single copy [26,27], while *B. subtilis AraA *and *E. coli AraD *where targeted to the rDNA region of *S. cerevisiae *to allow multiple copy integration [27].

Transformants were selected for their ability to grow on arabinose on yeast extract peptone arabinose (YPA) (see Methods section) plates. The rich medium provided conditions for the cells to recover from transformation. After 3 days, visibly larger colonies emerged over a confluent background of minute colonies growing on the rich medium. No larger colonies were detected on negative control transformation plates, even after several days of incubation. Strain TMB3073 was purified from 16 independently picked clones, which were re-streaked on yeast nitrogen base/arabinose (YNBA) plates. The presence of the integrated constructs was verified by polymerase chain reaction (PCR) with specific primers for *AraA*, *AraB *and *AraD *genes. This is to the best of our knowledge the first plasmid-free, arabinose growing *S. cerevisiae *laboratory strain.

Subsequently, two different xylose utilisation pathways were independently introduced in TMB3074. The XR and XDH pathway was introduced by transformation with plasmid pY7 [28], harbouring *XYL1 *and *XYL2 *encoding genes form *P. stipitis*, while the XI pathway was introduced by transformation with plasmid YEplacHXT-XIp [29], carrying a synthetic *Piromyces *sp. XI gene [29,38]. Persistence of the integrated arabinose pathway was re-confirmed with PCR. The two resulting strains, named XR/XDH and XI strain, respectively, are prototrophic and genetically identical, except for the plasmid-borne xylose utilisation pathways.

### Aerobic growth on defined pentose media

**Figure 2:**
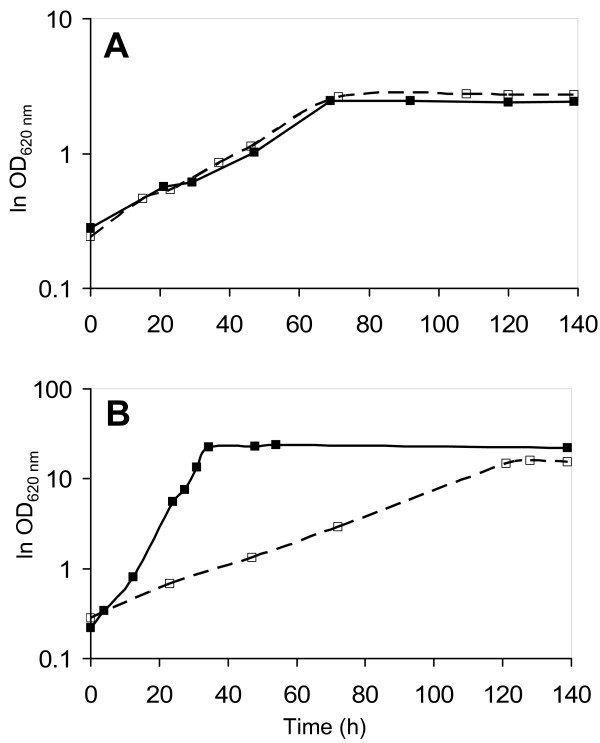
**Aerobic growth of *Saccharomyces cerevisiae *xylose reductase/xylitol dehydrogenase and xylose isomerase strains**. (A) Yeast nitrogen base/arabinose medium and (B) yeast nitrogen base/xylose medium. Solid line, filled symbols: xylose reductase/xylitol dehydrogenase strain; dashed line, open symbols: xylose isomerase strain.

The reciprocal effect of the two combinations of pentose utilisation pathways was first assessed under aerobic conditions. The XR/XDH and XI strains were grown in YNBA and YNB/xylose (YNBX) media (see Methods section) (Figure 2). The two strains exhibited similar growth patterns in the arabinose medium (Figure 2A), with calculated maximum specific growth rates of 0.03 ± 0.004 h^-1 ^for the XR/XDH strain and 0.03 ± 0.001 h^-1 ^for the XI strain. When xylose was provided as the sole carbon source, the XR/XDH strain grew at higher growth rate than the XI strain, with calculated growth rates of 0.14 ± 0.06 h^-1 ^for the XR/XDH strain and 0.03 ± 0.003 h^-1 ^for the XI strain (Figure 2B), which is in agreement with previously reported results [29].

### Anaerobic mixed sugar fermentation

**Figure 3:**
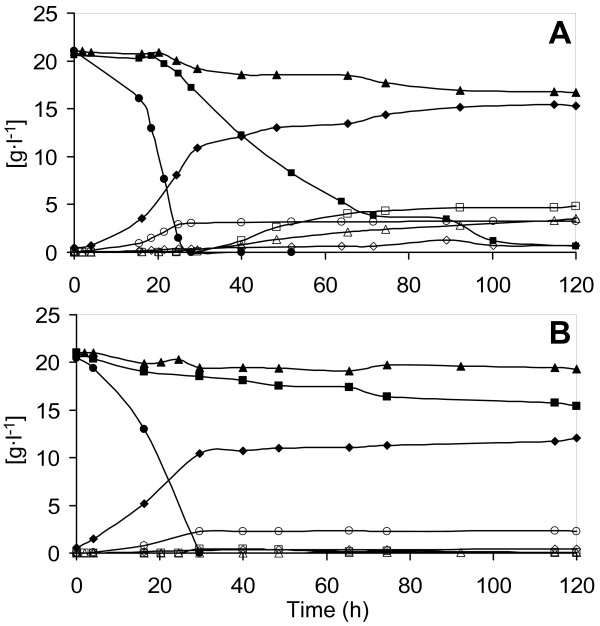
**Substrate and product concentration during anaerobic batch fermentation of defined mineral medium containing glucose, arabinose and xylose**. (A) Xylose reductase/xylitol dehydrogenase strain; (B) xylose isomerase strain. ●: glucose; ■: xylose; ▲: arabinose; ◆: ethanol; ◇: acetate; ○: glycerol; □: xylitol; △: arabitol.

Next, two strains were compared in anaerobic batch fermentation with a mixture of glucose, arabinose and xylose as a carbon source (Figure 3) (Table 2). Glucose was consumed first and completely exhausted at around 28–30 hours (Figure 3). Arabinose and xylose were co-consumed by both strains in the pentose phase, that is, the fermentation phase subsequent to glucose depletion, with xylose being consumed at a higher rate than arabinose by both strains. Biomass formation ceased after glucose depletion. Specific substrate consumption and product formation rates (Table 2) were calculated only for the pentose phase, when biomass was constant.

**Table 2:** Substrate consumption and product formation parameters during anaerobic batch fermentation of a mixture of glucose, arabinose and xylose in defined mineral medium

	**TMB3075 (xylose reductase/xylitol dehydrogenase)**	**TMB3076 (xylose isomerase)**
**Consumed arabinose **g l^-1^	4.04 ± 0.33	1.65 ± 0.01
**Consumed xylose **g l^-1^	20.1 ± 0.2	4.9 ± 0.7
q arabinose* (g arabinose) (g cells)^-1 ^h^-1^	0.014 ± 0.001	0.005 ± 0.001
q xylose* (g xylose) (g cells)^-1 ^h^-1^	0.09 ± 0.001	0.02 ± 0.001
**Final ethanol titre **g l^-1^	14.7 ± 0.5	11.8 ± 0.3
q ethanol* (g ethanol) (g cells)^-1 ^h^-1^	0.024 ± 0.001	0.010 ± 0.001
**Y ethanol, on total added sugars **(g ethanol) (g sugar)^-1^	0.23 ± 0.01	0.18 ± 0.01
**Y ethanol, on consumed sugars **(g ethanol) (g sugar)^-1^	0.32 ± 0.01	0.41 ± 0.01
**Y xylitol **(g xylitol) (g consumed xylose)^-1^	0.27 ± 0.04	0.04 ± 0.02
**Y arabitol **(g arabitol) (g consumed arabinose)^-1^	0.87 ± 0.03	ND
**Y biomass, on total added sugars ** (g dry cell weight) (g sugar) ^-1^	0.03 ± 0.01	0.03 ± 0.01
**Y biomass, on consumed sugars ** (g dry cell weight) (g sugar) ^-1^	0.04 ± 0.01	0.07 ± 0.01
**Y glycerol **(g acetate) (g consumed arabinose)^-1^	0.08 ± 0.001	0.09 ± 0.01
**Y acetate **(g acetate) (g consumed arabinose)^-1^	0.020 ± 0.002	0.016 ± 0.001

Values are the calculated average of two biological replicates. *Calculated after glucose depletion.

ND.: not determined. q: specific productivity, Y: yield.

The XR/XDH strain consumed 4.04 g l^-1 ^arabinose compared with 1.65 g l^-1 ^for the XI strain. The XR/XDH strain consumed nearly all xylose with an overall rate of 0.09 g (g cells)^-1 ^h^-1^. In contrast, only 8% of the xylose was consumed by the XI strain, at an overall rate of 0.02 g (g cells)^-1 ^h^-1^.

The XR/XDH strain produced a final ethanol concentration of 14.7 g l^-1^, with a yield based on total sugars of 0.23 g (g sugar)^-1^, while the XI strain reached an ethanol concentration of 11.8 g l^-1^, with a yield on total sugars equal to 0.18 g (g sugar)^-1 ^(Table 2). The specific ethanol productivity from pentose sugars for the XR/XDH strain was 0.024 g (g cells)^-1 ^h^-1^, versus 0.010 g (g cells)^-1^h^-1 ^for the XI strain (Table 2).

Almost 90% of the arabinose consumed by the XR/XDH-strain was converted to arabitol, whereas arabitol formation in the XI strain was essentially negligible. As a result, the final arabitol concentration was 3.51 g l^-1 ^for the XR/XDH strain, whereas it was below detection for the XI strain. At the same time, the XR/XDH strain converted part of the consumed xylose to xylitol, with a yield of 0.27 g xylitol per gramme of consumed xylose. The lower by-product formation gave the XI strain a higher ethanol yield on consumed sugars, 0.41 (g ethanol) (g consumed sugar)^-1^, compared with 0.32 (g ethanol) (g consumed sugar)^-1 ^for the XR/XDH strain.

Biomass was formed with a yield of 0.032 g g^-1 ^calculated on total sugars, with a final dry cell weight of 1.95 g l^-1 ^for both strains. Acetate is usually generated by *S. cerevisiae *in response to a demand for NADPH. This can be associated with the need of this cofactor for biosynthetic reactions. In the XR/XDH strain, NADPH demand might be higher due to the utilisation of this cofactor by the XR NADPH requirement. Consistently, acetate yield was slightly but significantly higher in the XR/XDH strain, while the opposite was observed for glycerol yield.

## Discussion

To the best of our knowledge, arabinose and xylose co-metabolism in recombinant *S. cerevisiae *strains has previously only been reported for one recombinant industrial strain of *S. cerevisiae *[27]. In this strain, the XR/XDH pathway was combined with a functional bacterial arabinose utilising pathway [26]. While arabinose and xylose were co-consumed, only xylose was further metabolised to ethanol. Arabinose was instead converted to arabitol, which was believed to be catalysed by the overexpressed heterologous *P. stipitis *XR enzyme. In fact, this enzyme has a lower *K*m for arabinose than for xylose [39]. Arabitol is a known inhibitor of arabinose isomerase [40] and the results suggested that arabinose metabolism in this recombinant *S. cerevisiae *strain was limited by arabitol inhibition of the first enzyme in the pathway.

The current investigation aimed to explore whether arabitol formation during co-utilisation of arabinose and xylose could be avoided or minimised when xylose metabolism was instead governed by an isomerase pathway (Figure 1). This would allow co-utilisation of arabinose and xylose under conditions where arabitol formation and inhibition of arabinose isomerase was reduced or absent. In fact, arabitol formation was completely abolished when the two isomerase pathways were combined in one strain, TMB3076 (Table 2), confirming that arabitol formation was caused by overexpression of the heterologous XR. However, the absence of arabitol formation was the only benefit observed during arabinose and xylose co-utilisation by the isomerase strain. The XR/XDH strain was superior in all other aspects, that is, sugar uptake rate, ethanol concentration, ethanol yield on total sugars and ethanol productivity.

Heterologous expression of xylose and later, arabinose isomerase in *S. cerevisiae *have been very tedious undertakings. Already suggested in the late 1970s, it required another 16 years before the first XI was functionally expressed in *S. cerevisiae *[28]. Furthermore, it was only when XI expression from multi-copy plasmids was combined with extensive adaptation protocols that a functional xylose fermenting recombinant *S. cerevisiae *strain was obtained [41]. Similarly, the first arabinose fermenting recombinant *S. cerevisiae *strain had also undergone extensive adaptation in addition to being transformed with genes for the bacterial arabinose pathway [26]. The same results were later obtained with an arabinose pathway based on other bacterial genes [42]. While the beneficial effects of these extensive adaptation protocols remain to be clarified, it is tempting to speculate that heterologous isomerases may not be able to express their full catalytic potential, in terms of substrate conversion rate, in the *S. cerevisiae *intracellular environment.

In addition to adaptation, codon optimisation has been shown to improve the performance of heterologous bacterial isomerase pathways in *S. cerevisiae *[43]. Both ethanol yield and specific ethanol productivity were significantly increased when the codon usage of the bacterial genes was adapted to the usage of glycolytic yeast genes.

In *S. cerevisiae*, pentose sugars are reduced to the corresponding pentitols by the unspecific AR encoded by *GRE3 *[33,34] as well as by a number of uncharacterised open reading frames [44]. This was well illustrated by an arabinose-adapted recombinant strain of *S. cerevisiae *carrying a *GRE3 *deletion [42]. This strain reduced xylose to xylitol most likely through one of its unspecific reductases, while arabinose reduction to arabitol formation was negligible. However, despite the presence of a highly expressed *xylA *gene encoding XI, the strain was incapable of xylose growth.

In the XR/XDH strain, arabitol formation represents a dead end in the metabolism, since yeast lacks the enzymatic activity to further convert arabitol. In addition, arabitol has been extensively reported to be a potent inhibitor of arabinose isomerase [36,40,45-48], which was illustrated by the observation that the highest arabinose consumption rate occurred between 25 and 40 hours (Figure 3A), when glucose was depleted and prior to arabitol build-up. Thus, the XR/XDH strain displays a lower yield on consumed sugars, but the higher consumption rates of pentose sugars compensate for this, resulting eventually in higher specific ethanol productivity.

## Conclusion

The combination of the XR/XDH pathway and the bacterial arabinose isomerase pathway resulted in both higher pentose sugar uptake and higher overall ethanol production than the combination of the XI pathway and bacterial arabinose isomerase pathway. Moreover, the flux through the bacterial arabinose pathway did not increase when combined with the XI pathway. This suggests that the low activity of the bacterial arabinose pathway cannot be ascribed to arabitol formation via the XR enzyme.

## Methods

### Strains and media

Yeast strains and plasmid utilised for this work are summarised in Table 1. *E. coli DH5α *(Life Technologies, Rockville, MD, US) was used as an intermediate host for cloning steps and plasmid amplification and was routinely grown in lysogeny broth medium [49] containing 100 mg l^-1 ^ampicillin (Shelton Scientific, Shelton, CT, US). *S. cerevisiae *strains were grown aerobically in YNB (Difco Laboratories-Becton, Dickinson and Co., Sparks, NV, US), buffered at pH 5.5 with 50 mM potassium hydrogen phthalate (Merck, Darmstadt, Germany) [50] and formulated as it follows: YNB/glucose 20 g l^-1 ^glucose, 6.7 g l^-1 ^YNB; YNBA 50 g l^-1 ^arabinose, 13.4 g l^-1 ^YNB; YNBX 50 g l^-1 ^xylose, 13.4 g l^-1 ^YNB. According to strain requirements, the medium was supplemented with uracil and/or leucine at concentrations of 40 mg l^-1 ^and 240 mg l^-1^, respectively. YPA medium for initial selection of arabinose-growing strains was composed of 10 g l^-1 ^yeast extract (Merck, Darmstadt, Germany), 20 g l^-1 ^peptone (Merck, Darmstadt, Germany), 50 g l^-1 ^arabinose (Sigma-Aldrich, St Louis, MO, US). Solid media were obtained by addition of 20 g l^-1 ^agar (Merck, Darmstadt, Germany).

Anaerobic mixed sugar batch fermentation was performed in defined mineral medium [51] supplemented with 0.4 g l^-1 ^Tween 80 (Sigma-Aldrich, St Louis, MO, US), 0.01 g l^-1 ^ergosterol (Alfa Aesar, Karlsruhe, Germany), 20 g l^-1 ^glucose (VWR International, Poole, UK), 20 g l^-1 ^xylose (Acros Organics, Geel, Belgium) and 20 g l^-1 ^arabinose (Sigma-Aldrich, St Louis, MO, US).

### Nucleic acid manipulation

Standard molecular biology techniques were used [49]. The lithium acetate/dimethyl sulphoxide protocol was used for yeast transformation [52]. Yeast chromosomal DNA was extracted with a bead-beater (Biospecs Products, Bartlesville, OK, US) and phenol/chloroform [49]. Plasmid DNA was purified from *E. coli *with Gene JET plasmid miniprep kit (Fermentas, St Leon-Rot, Germany). Linear integration cassettes were PCR-amplified from plasmid prDNAAraA and prDNAAraD [27] with PWO DNA polymerase (Fermentas, St Leon-Rot, Germany) using the following thermal cycler programme: 94°C 5 min; 25 cycles of 94°C 30 s, 46.5°C 30 s, 72°C 3 min; final extension 72°C 7 min. The two PCR reactions were treated with DpnI endonuclease (Fermentas, St Leon-Rot, Germany) for the removal of template plasmid DNA, pooled, precipitated with ethanol and resuspended in 15 μl of TE buffer (10 mM Tris, 1 mM ethylenediaminetetraacetic acid, pH 8.0), which was used for yeast transformation. Analytical PCR was performed with Taq DNA polymerase (Fermentas, St Leon-Rot, Germany).

### Cultivation conditions

*S. cerevisiae *was grown aerobically in 1 litre baffled flasks containing 0.1 litre medium, incubated at 30°C in a rotary shake-incubator (INR-200 Shake Incubator, Gallenkamp, Leicester, UK) at 200 rpm. Cultures were inoculated at an initial optical density (OD)_620 nm_of 0.2 ± 0.02 with sterile H_2_O-washed cells from a late-exponential YNBG pre-culture. The maximum specific growth rate, μ, was calculated from exponential fitting of growth curves from at least two biological duplicates.

Anaerobic mixed-sugar batch fermentation was performed in 1.5 litre working volume bioreactors (Applikon Biotechnology, Schiedam, The Netherlands), for 120 hours, at 30°C, 200 rpm at pH 5.5 automatically controlled by the addition of 3 M potassium hydroxide. Anaerobic conditions were established prior to inoculation by sparging the medium for at least 3 hours with nitrogen (< 5 ppm oxygen) (AGA, Malmö, Sweden) at 0.2 litre/min flow rate. Nitrogen was sparged throughout the fermentation at the same flow rate. Cells were pre-grown aerobically in shake flasks in YNBG medium, harvested by centrifugation, resuspended in about 10 ml sterile medium and inoculated in the fermentor at an initial OD_620 nm _of 0.2. Fermentation experiments were performed in duplicate.

### Analyses

Samples were drawn from the fermentors after discharging the sample tubing dead-volume, cells were quickly separated by centrifugation and the supernatant was filtered through 0.20 μm membrane filters (Toyo Roshi Kaish, Tokyo, Japan) and stored at 4°C until further analysis.

Concentrations of glucose, xylose, acetate, glycerol and ethanol were determined by high performance liquid chromatography (HPLC) (Beckman Instruments, Fullerton, CA, US). The compounds were separated with three Aminex HPX-87H resin-based columns (Bio-Rad, Hercules, CA, US) connected in series and preceded by a Micro-Guard Cation-H guard column (Bio-Rad, Hercules, CA, US). Separation was performed at 45°C, with 5 mM sulphuric acid at a flow rate of 0.6 ml/min as mobile phase. Owing to evaporation, ethanol concentrations were calculated from the degree of reduction balance of the overall carbon stoichiometry of the fermentation.

Concentrations of arabinose, arabitol and xylitol were determined by HPLC (Waters, Milford, MA, US) using an HPX-87P resin-based column (Bio-Rad, Hercules, CA, US) preceded by a Micro-Guard Carbo-P guard column (Bio-Rad, Hercules, CA, US). Separation was performed at 85°C, with water at a flow rate of 0.5 ml/min as mobile phase. All compounds were quantified by refractive index detection (Shimadzu, Kyoto, Japan). For each HPLC run, a seven-point calibration curve was made for each compound to calculate concentrations. Each sample was analysed at least in duplicate and a maximum of 10% difference between replicate analyses was accepted.

For each fermentation experiment, dry weight measurements were made in three points at least, in triplicate for each point. The end point of the fermentation (*t *= 120 h) was always included. For dry weight determination, a known volume of cell culture was filtered through dry pre-weighed 0.45 μm nitrocellulose filters, which were subsequently dried in a microwave oven and weighed.

## Abbreviations

AR: aldose reductase; HPLC: high performance liquid chromatography; NADH: nicotinamide adenine dinucleotide; NADPH: nicotinamide adenine dinucleotide phosphate; OD: optical density; PCR: polymerase chain reaction; PPP: pentose phosphate pathway; XDH: xylitol dehydrogenase; XI: xylose isomerase; XK: xylulokinase; XR: xylose reductase; YNB: yeast nitrogen base; YNBA: yeast nitrogen base/arabinose; YNBG: yeast nitrogen base/glucose; YNBX: yeast nitrogen base/xylose; YPA: yeast extract peptone arabinose

## Authors' contributions

MB participated in the design of the study, performed the experimental work and wrote the manuscript. BHH participated in the design of the study and commented on the manuscript. MFGG participated in the design of the study and commented on the manuscript. All the authors read and approved the final manuscript.
